# A review on the achievement of enzymatic glycerol carbonate production

**DOI:** 10.55730/1300-0527.3445

**Published:** 2022-04-05

**Authors:** Selda AYDOĞDU, Nurcan KAPUCU

**Affiliations:** Department of Chemical Engineering, Faculty of Engineering, Kocaeli University, Kocaeli, Turkey

**Keywords:** Glycerol carbonate, biofuel additive, glycerol, dimethyl carbonate, biodiesel, enzymatic transesterification

## Abstract

High energy demand driven by decreasing fossil fuels, and global warming because of the burning of fossil fuels necessitates the utilization of renewable and clean energy. One of these renewable energy sources is biodiesel. The increasing trend of biodiesel over the last 20 years tends to result in increasing glycerol (Gly), which is produced during the biodiesel production in 10% ratio (w/w) as a by-product. Using Gly as raw material is an alternative way to produce bio-based new products such as glycerol carbonate (GlyC). GlyC is a value-added product of Gly/vegetable oil and this product can be used as a potential fuel additive in the future because of its high oxygen content. Furthermore, GlyC shows a high reactivity; therefore, it is a bio-based building block for complex chemicals. The present review gives information about the bio-catalytic synthesis of GlyC from Gly/vegetable oil and, dimethyl carbonate (DMC). In addition, the influencing parameters of this bio-catalytic GlyC synthesis are discussed and reviewed in this paper.

## 1. Introduction

The major energy demand in the world is covered by fossil fuels (81.1%), with oil (31.8%), coal (27.1%), and natural gas (22.2%), while biofuels and waste fuel provide for 9.5% of the total primary energy consumption [1]. Consumption of fossil fuels causes environmental pollution and global warming. After the oil crisis in the early 1970s, several governments were encouraged to explore alternative energy fuel sources. In 1975, the Brazilian Government initiated replacing gasoline with bioethanol, which is produced from sugarcane. With this action, biofuels started as a serious option to petrol [2]. The demand for fossil fuels and air pollution because of their combustion put forward biofuel using as alternative clean energy resources. Biodiesel is one of the biofuels.

Energy Information Administration projects that between 2020 and 2050, the energy consumption will increase by approximately 50% [3] and the carbon dioxide (CO_2_) emission will grow approximately 20% [4]. The emission during the combustion of fossil fuels such as petroleum products and coal brings on air pollution, greenhouse effect, and global warming [5–7]. The combustion of biodiesel creates less CO_2_, sulfur dioxide (SO_2_), hydrocarbon emission, and particulate matter [8–9].

Biodiesel is produced through transesterification of animal fats or vegetable oils with methanol in the presence of an acid, alkali catalyst, or biocatalyst [10–14]. During biodiesel production, Gly is also produced as a by-product of about 10% (percentage by weight) [15–18]. Biodiesel can also be synthesized by noncatalytic conditions in supercritical conditions [19,20]. The disadvantages of these supercritical methods are demand for high pressure and high temperature [21]. Biodiesel can also be produced from vegetable oil and dialkyl carbonate (e.g., DMC) without Gly production. During biodiesel production with DMC, GlyC is produced as a by-product [22]. Increased Gly production lowers the unit price of Gly. The global Gly market size was about USD 2.6 billion in 2019 and it is expected that the annual Gly growth rate will increase 4% from 2020 to 2027 [23]. Gly is flammable, insoluble, and has low volatility in hydrocarbon-derived liquid fuels [24]. Gly is polymerized at high temperatures and oxidized partially to the toxic acrolein. The burning of Gly causes corrosion of diesel motor [25] and clog of the engine; therefore, it is not suitable as a fuel or fuel additive [26]. On the other hand, Gly derivatives such as ethers, acetyls/ketals, and esters, etc. can be mixed with fossil-origin fuels without a phase change. This actuates the researcher’s discovery to convert low-efficient Gly to value-added products based on chemicals, chemical intermediate, fuel, and fuel additives [27,28]. One of the promising and potential methods is the transforming of Gly into an oxygenated fuel additive for the automotive and biorefinery industries. Widely used oxygenated fuel additives are alcohol, ether, and ester functional groups [29]. The oxygenated fuel additive causes reduction of greenhouse gas (GHG) emissions compared to gasoline [30] and offers complete fuel combustion in the combustion chamber [8].

**Figure 1:**
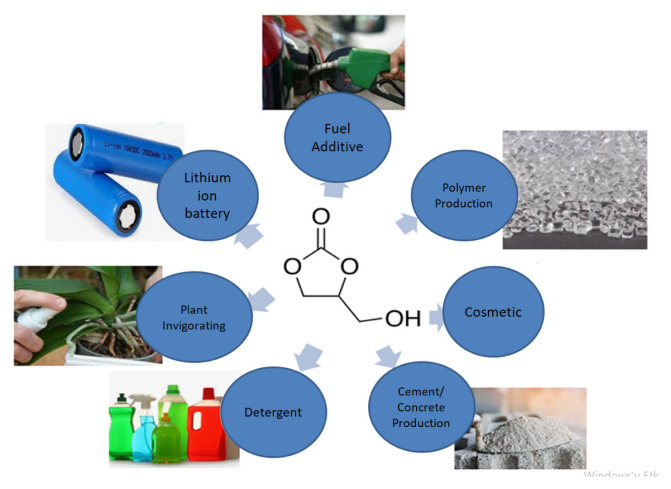
Applications of GlyC.

GlyC is a value-added product of Gly. GlyC is biodegradable, water-soluble, nontoxic, and viscose. GlyC is included in the carbonate ester group and has high oxygen content (54.2 %) in comparison to other organic carbonates (DMC, diethyl carbonate (DEC), propylene carbonate (PC), butylene carbonate (BC)) [31]. Thanks to its high oxygen content, the GlyC has the potential for use as a fuel additive and can reduce hydrocarbons, CO_2_, particulate matter in fuel and contribute to clean combustion [28]. The use of 2%–4% addition of carbonate ester in diesel reduces the soot emission up to approximately 50% [32]. GlyC offers a wide reactivity because of both hydroxyl groups and the 2-oxo-1,3-dioxolane group on its molecular structure. Additionally, GlyC can be used directly as an additive in the cosmetic, liquid gas separation process, detergent, plant invigorating, lithium/lithium-ion batteries, and cement or concrete production. It is used indirectly in polymer production, resin, and surface-active material production [33]. Figure 1 shows applications of GlyC. The global market value of GlyC amounted to US $1.17 B in 2020 and it will increase US $1.80 B in 2027. During COVID-19, the shutdowns had disruptive effects on the battery value chain. GlyC is an important multifunctional monomer for the production of polycarbonate and polyurethanes [34] and also for lithium batteries. The growing demand for lithium batteries from various industries has driven the growth of the GlyC market over the forecast period. In 2020, Asia held the highest share (56.24%) in the GlyC market. In countries such as China, Japan, Korea, Thailand, Taiwan, Singapore, and Malaysia, polycarbonate production from GlyC has increased. In Asia, the growing demand for lithium-ion batteries for the electronics and automotive industries is expected to expand GlyC market [34]. Two key world market players of GlyC producers are Huntsman and Ube [35]. GlyC is produced by Huntsman from PC and Gly by an ester change reaction in presence of a chemical catalyst at the temperature of 100–150 °C and 35 mmHg [36] with 93 % (wt) GlyC conversion [37]. The company Ube produces GlyC by Gly and DMC with the potassium carbonate at the temperatures of 70–120 °C with 97% (wt.) GlyC conversion [38].

Enzyme catalysis is accepted as mild (low temperature and pressure) and eco-friendly, and lipases do not require a solvent for the GlyC synthesis. Enzyme reusability and recovery of the desired product are easy. The enzymatic catalysis requires less energy consumption compared to chemical catalysis. The product separation and purification processes are simple [39]. Such properties of enzymes make the enzymatic GlyC synthesis attractive in the context of a greener way.

Although there is no direct study of enzymatic review in the literature, there are some studies of general production belonging to GlyC production, in which the enzymatic production has been briefly mentioned [33,39]. Kim et al. investigated an enzymatic GlyC production with Novozym 435 under the reaction conditions; 60 °C, 1:1 DMC: Gly molar ratio, 28 h with the solvent tetrahydrofuran (THF), and a GlyC conversion of 100% was obtained. Nascimento et al. researched enzymatic transesterification reaction of GlyC production with vegetable oil and DMC by epoxy immobilized *Candida antarctica* lipase (CalB) and *Porcine pancreas* lipase (PPL), under continuous flow conditions of 60 °C, 10:1 DMC: vegetable oil molar ratio, in 90 min reaction time with the solvent methyl tert-butyl ether (MTBE); a GlyC conversion of 99% and a GlyC selectivity of 99% were reached.

The main objectives of this paper are to review the latest developments in the field of the lipase-catalyzed GlyC transesterification process from DMC and Gly/vegetable oil and to describe the advantage of the potential using of GlyC as a fuel additive and as a building block for different chemicals. Also, another objective is to reveal the parameters affecting GlyC production, which is an alternative, environmentally friendly, and greener route compared to chemical homogeneous/heterogeneous catalysts with mild reaction conditions.

## 2. GlyC production routes from Gly

**Figure 2:**
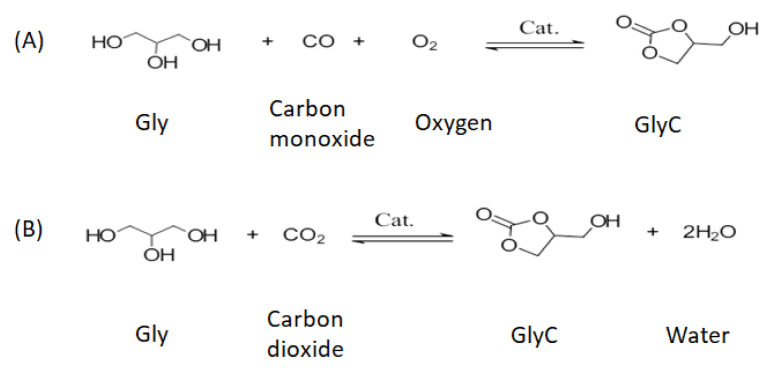
Synthesis of GlyC with CO (A) and CO_2_ (B).

**Figure 3:**
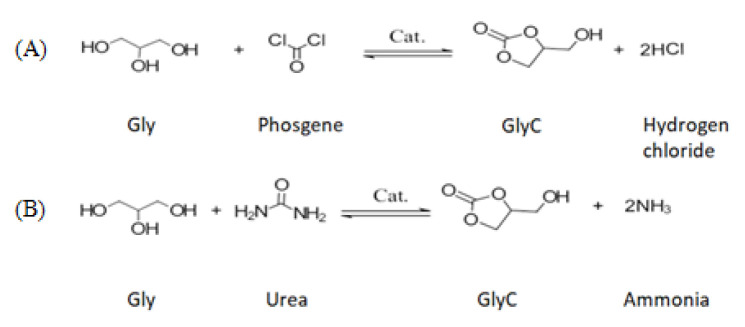
Synthesis of GlyC with phosgene (A) and urea (B) from Gly.

**Figure 4:**
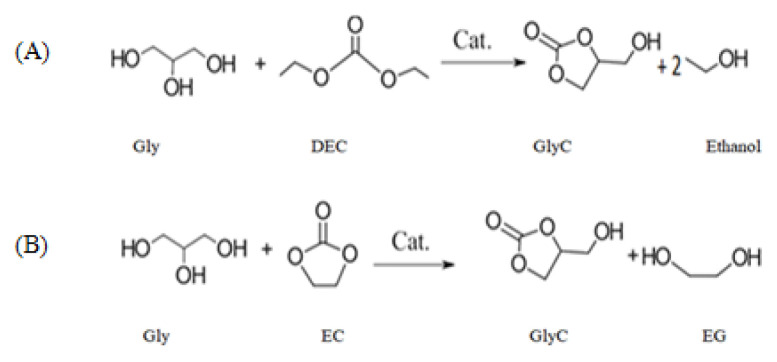
Synthesis of GlyC with DEC (A) and EC (B).

**Figure 5:**
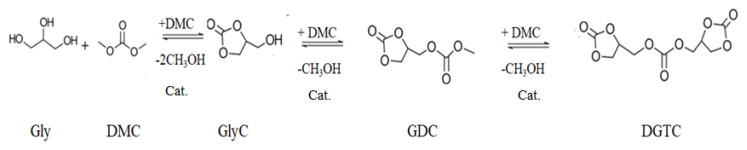
Synthesis of GlyC with DMC and Gly. (GDC: Glycerol dicarbonate, DGTC: Diglycerol tricarbonate)

GlyC production from Gly can be achieved through both carbonation and transcarbonation reactions. In a carbonation reaction, carbon monoxide (CO) and CO_2_ react with the Gly to GlyC [33,40]. The carbonation reactions with CO and CO_2_ are presented in Figures 2A and 2B, respectively [33,41]. The disadvantages of carbonation reactions are that this reaction with CO and CO_2_ includes unsafe working conditions; necessity of an organic solvent, high reaction temperature, high pressure, long response time, and recovery problems of chemical catalysts [33]. Additionally, CO is toxic and inflammable [42]. An alternative to carbonation reaction is transcarbonation, in which a carbonate exchange reaction between alcohols and carbonate groups occurs. Carbonate exchange from one carbonate to another follows a nucleophile attack between the carbon atom of carbonate groups and the oxygen atom of the hydroxyl group of the alcohol. As carbonate sources, three different groups, i.e. phosgene, urea, and carbonate derivates (DEC, ethylene carbonate (EC), and DMC) were submitted. The reactions of phosgene [43] and urea [41–43] with Gly by a chemical catalyst are presented in Figures 3A and 3B, respectively [33]. Phosgene is a toxic reactant, so the use of phosgene as a reactant is very restricted. The reaction of Gly with urea produces ammonia as a by-product, which reduces carbonate conversion of Gly [33]. A safer alternative, transcarbonation reaction, involves other carbonate derivatives as well as DEC [44], EC [45], and DMC [46]. The reactions of DEC and EC with Gly are presented in Figures 4A [47] and 4B [45], respectively. DMC reaches a higher GlyC conversion compared to DEC [48]. EC is an expensive carbonate group and by-product EG has high boiling point, which makes the purification step difficult [49]. For this reason, DMC is the most preferred acyl acceptor for using GlyC production. In Table 1, the advantages and disadvantages of different carbonate derivates are presented, which is used in the production of GlyC. DMC is becoming advantageous compared to the other carbonate derivatives because of their high chemical reactivity, lower boiling point (90 °C), nontoxic, and biodegradable properties [50–53]. Therefore, in the GlyC production, DMC is considered. In Figure 5, the transesterification reaction of GlyC from DMC and Gly is presented. Gly and DMC create a short-life intermediate and methanol. Afterward, this short-life intermediate is transformed into an intramolecular cyclization (GlyC) and methanol. By-products of this reaction are glycerol dicarbonate (GDC) and diglycerol tricarbonate (DGTC). Side products formation is affected by reactant molar ratio (DMC:Gly), enzyme type, and temperature [54].

Enzymatic GlyC synthesis with Gly and DMC is the most direct and industrially operable way with a high GlyC yield [55]. In the next section of this review, the enzymatic synthesis from DMC will be systematically discussed.

## 3. Enzymatic transesterification of Gly/vegetable oil to GlyC with DMC

Enzymatic GlyC production from Gly and DMC is an alternative and environmentally friendly catalysis route, which is performed by lipases. For the enzymatic GlyC production, different free and immobilized lipases from different sources are utilized. GlyC can be produced with enzymes such as CalB in free form [48,54,56], CalB in immobilized form (commercial name: Novozym 435) [49,55–59], *Aspergillus niger* (*A.niger*) lipase in free form [60] and in immobilized form [61,62]. In Table 2, the enzymatic GlyC transesterification reaction conditions; utilized catalyst, catalyst loading, reactant molar ratio, source, solvent/solvent-free medium, temperature, reaction time, and GlyC conversion/yield/selectivity from DMC, and Gly are submitted. The GlyC synthesis from Gly and DMC has been successfully (with 99% conversion) performed using Novozym 435 in presence of solvent THF. The stoichiometric molar ratio of reactants (DMC: Gly) was 1:1 with a reaction time of 28 h [57]. The enzymatic GlyC production can also be investigated in solvent-free conditions with excess DMC, which is researched by Tudorache (2017) with magnetic nanoparticle immobilized *A. niger lipase.* The reaction conditions were 60 °C, 10:1 DMC: Gly molar ratio, 6 h reaction time, solvent-free; and a Gly conversion of 87.7% was achieved.

GlyC can also be produced by vegetable oil and DMC as a by-product during Gly-free biodiesel production. The advantage of Gly-free production with DMC is that lipase is not inactivated by short-chained alcohol (e.g., methanol), which is used in standard biodiesel reactions.

**Figure 6:**
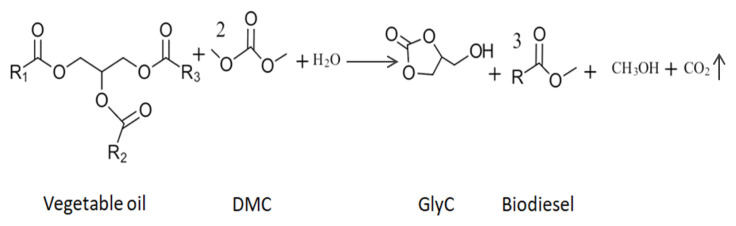
Synthesis of GlyC with DMC and vegetable oil.

**Figure 7:**
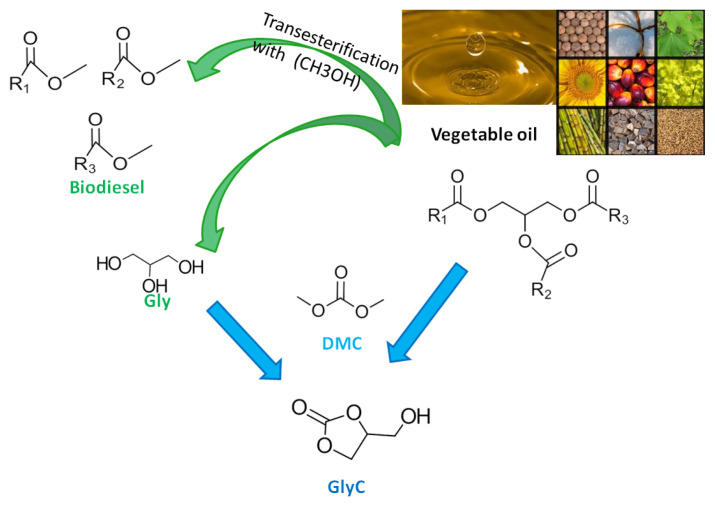
Synthesis route of GlyC from DMC.

The reaction of vegetable oil and DMC to GlyC and biodiesel is irreversible and consists of 3 cascade reactions. In the first cascade reaction, vegetable oil and DMC react to form biodiesel and fatty acid glycerol carbonate monoesters (FAGCs). In the second cascade reaction FAGCs and DMC form biodiesel and GDC. In the last cascade reaction in presence of water, GDC is transformed to GlyC, methanol, and CO_2_. In Figure 6, the reaction of 3 cascade reactions is summarized and the transesterification reaction of vegetable oil and DMC to produce GlyC and biodiesel is presented. Leão et al. (2016) investigated the enzymatic GlyC production by Accurel immobilized CalB (CalBAcc) both with Gly and vegetable oil as reactant. They researched enzymatic GlyC production in batch conditions at 60 °C, 2:1 molar ratio of DMC: Gly, 20% enzyme loading (CalBAcc), and 48 h reaction time. There was a GlyC conversion of 80%. They also researched the enzymatic GlyC synthesis from vegetable oil (soybean oil, palm oil, and macaúba oil) and DMC under batch conditions. Reaction conditions were 60 °C, 1:10 molar ratio of vegetable oil: DMC molar ratio, 20% enzyme loading (CalBAcc), 0.7% water (v/v) and 48 h reaction time. For all of the vegetable oils, there was a GlyC conversion/selectivity of 99%. More GlyC conversion was achieved with vegetable oil [55]. In Table 3, the enzymatic GlyC transesterification reaction conditions, and GlyC conversion/yield/selectivity from DMC and vegetable oil are given. In Figure 7, the GlyC synthesis route from Gly/vegetable oil and DMC is presented. GlyC can be directly obtained from vegetable oil with DMC or from Gly (a by-product of biodiesel) with DMC produced. In Section 4, the catalytic mechanism of lipase for enzymatic GlyC production is described.

## 4. Catalytic mechanism of enzymatic transesterification reaction for GlyC production

**Figure 8:**
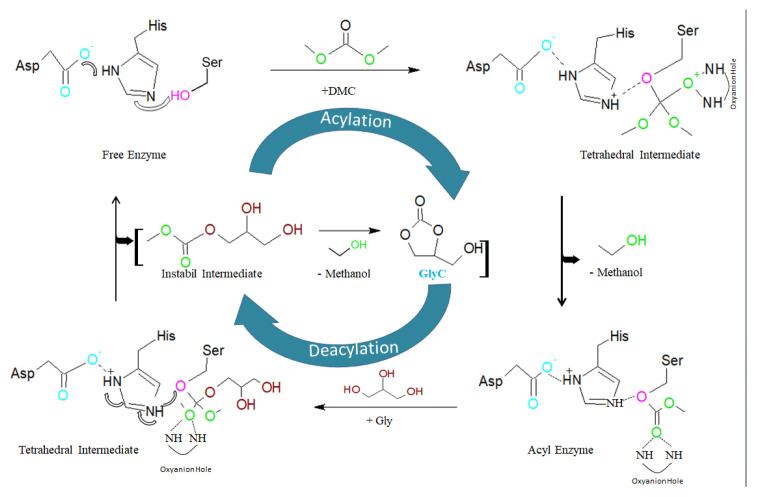
Catalytic mechanism of lipase for GlyC production.

Enzyme catalyzed reaction with lipase takes place in the active site, and the active sites of lipase have different shapes, sizes, depths, and geometry [63]. An active site consists of serine (Ser), histidine (His), and aspartate (Asp) triad and an oxyanion hole [64,65]. This triad of the active site acts as a charge relay system [66]. Most lipases have a lid domain formed α-helix, which is controlling access to the active site [67]. By the movement of the α-helical lid around the catalytic triad at the oil-water interface, the lipase is activated [68,69]. In the lipid-water interface, the lipase lid opens and the lipase is enabled to work effectively [70]. In Figure 8, the catalytic mechanism of lipase during GlyC production is presented. The mechanism starts with the acylation step, in which the proton is carried between the serine, histidine, and aspartate triad, and the hydroxyl group of serine is activated [71]. The carboxylate group on aspartic acid is bonded on the hydrogen of histidine, and the nitrogen atom of histidine is bound on the hydrogen of alcohol on serine. Hydroxyl group of serine attacks the ester binding of DMC in order to form a tetrahedral intermediate, which is stabilized by the oxyanion hole (Gln, Thr). In the next step, the ester bond is divided and methanol is removed, to form an acyl-enzyme intermediate. In the last step, in the deacylation process, the acyl-enzyme attacks nucleophilic to Gly by the catalytic histidine and aspartic acid. Finally, the short-life intermediate is formed, which is unstable and it is formed to GlyC by intramolecular cyclization process and the methanol occurs as by-product at the same time. In Section 5, the parameters that affect the enzymatic GlyC transesterification from DMC and Gly/vegetable oil are described.

## 5. Factors influencing enzymatic transesterification reaction in the GlyC production

The synthesis of GlyC depends on the reaction parameters such as reaction temperature, raw material type and amount, solvent addition, enzyme type, enzyme loading, support type, form of the enzyme, reactor type, process classification, reaction time, impurities, and reusability of the enzyme. In this section, the influencing parameters of enzymatic GlyC production are described in detail.

### 5.1. Effect of temperature

The temperature has a direct effect on reaction rate, which is related to enzyme stability, reagent solubility, and reaction kinetic constants. During the increasing of temperature, two conflicting mechanisms occur. With increasing temperature, the enzyme activation increases because of the increasing rate constant. On the other hand, increasing temperature causes denaturation of the enzyme (the thermal unfolding of enzyme quaternary and tertiary structures). Every enzyme has an optimum temperature, at which the greatest amount of substrate changes in the time units [72]. According to Arrhenius models, the reaction rate raises with increasing temperature and activation energy so that the reaction temperature affects directly the reaction rate [73]. The activation energies of enzyme-catalyzed reactions range between 4 and 20 kcal/g mole [68]. The activation energy of enzymatic GlyC production from palm oil and DMC was calculated about at 18.96 kJ/g mole (4.54 kcal/g mole) at 40 °C [69]. Panadare et al. (2016) investigated the effect of temperature on the reaction rate constant. The reaction rate constant is increased from 0.0041 (323K) to 0.0061(343K). The optimum temperature of GlyC transesterification reaction is varied depending on the immobilization support, enzyme type, catalyst loading, DMC:Gly/vegetable oil, and solvent amount. As Table 2 presents, optimum GlyC production by enzymatic transesterification can be achieved at a temperature range between 50 °C and 70 °C. The temperature is a directly dominant factor for GlyC yield. Li and Wang found out that the chemical equilibrium constant of GlyC reaction from Gly and DMC increases with rising temperature from 30 °C (0.221) to 70 °C (0.239) [41]. Min and Lee investigated the effect of temperature (40 °C, 60 °C, and 85 °C) on GlyC production from corn oil and DMC by Novozyme 435 (10% w/w), in 0.2% (v/v) water and 45 h. They found out, that the optimum temperature for enzymatic GlyC production using Novozym 435 was 60 °C [116]. In enzymatic GlyC production, the enzyme cannot dissolve in the reaction medium at lower temperature (40 °C), because of the high viscosity of the medium. Gly has a viscose liquid and has a decisive effect of the reaction medium. If the temperature of Gly rose from 302.59 K (30 °C) to 333.15 K (60 °C), the viscosity decreased from 648.4 mPa.s to 84.20 mPa.s [75]. With the rising temperature, the viscosity of the reaction medium reduces and the collision rate of reactants increases [49]. Lanjekar and Rathod analyzed the temperature effect (40–70 °C) on GlyC conversion with the following reaction conditions 1.5:1 DMC:Gly molar ratio, 400 rpm, 22% biocatalyst (w/w based on total Gly and DMC amount) and tert-butanol as solvent. The optimum temperature was 60 °C with a Gly conversion of 94.85% in 14 h [49]. At a temperature greater than 70 °C, the enzyme is deactivated because of the disruption of the active conformation of the enzyme, causing a decrease of enzyme activity (60 °C (8.75 U/g), 70 °C (6.27 U/g)) [49].

### 5.2. Effect of raw material amount

GlyC can be produced from the reactants Gly/vegetable oil (waste oil, microalgae oil) with DMC [76]. In this transesterification reaction, the Gly/vegetable oil is used as an acyl donor and DMC is used as a carbon source and acyl acceptor. The transesterification reaction of GlyC from DMC and Gly is the most commonly used and direct method and this reaction is irreversible. Theoretically, according to stoichiometry, to produce 1 mol of GlyC an equimolar mixture of substrates is needed; 1 mol of DMC and 1 mol of Gly. On this reaction, 2 mol of methanol is produced as a by-product as shown in Figure 4. High Gly concentration can lead to the coat onto the surface of the immobilized enzyme [77], which limits substrate and product diffusion [78], and subsequently, negatively affects the lipase activity [79] and process stability [80]. To shift this chemical equilibrium to the right side, towards GlyC, a high molar ratio of DMC to Gly is required [33]. In general, the enzymatic transesterification reaction is performed in high reagents ratio (DMC: Gly molar ratio in a range of 2–10) [66,68–70] There is much research about the conversion of pure Gly to GlyC. Tudorache et al. researched the enzymatic GlyC synthesis by waste glycerol (W-Gly) or with pure Gly [80–82].

The enzymatic GlyC production can also be produced by vegetable oil and DMC (See Figure 5). During standard biodiesel production by short-chained alcohol (methanol), the enzyme can be deactivated [83] and by-product Gly during biodiesel production can lead to mass transfer limitations and reaction rate reduction [14] and it can also inhibit the transesterification reaction by rapid deactivation of lipase [73]. GlyC can be produced as a by-product from vegetable oil and DMC during biodiesel production. This way of Gly-free production of biodiesel is an irreversible transesterification reaction. GlyC can be easily separated by distillation from biodiesel after the reaction [62]. The high stoichiometric ratio of acyl acceptor to oil raises the reaction rate and increases the conversion to biodiesel and GlyC. Another advantage of excess DMC is the decrease of collision between oil and lipase because of diluted concentration [84].

To our knowledge, there is no research article about the enzymatic GlyC production using waste edible oil directly. Using waste cooking oil can decrease the feedstock cost of GlyC production. In comparison to the terrestrial plant, the growth rate of the microalgae is higher. Microalgae oil content is 20%–80% of dry biomass [85]. These oils would also be alternative options to vegetable oils and waste edible oils. For simultaneous preparation of biodiesel and GlyC, Novozym 435 catalyzed reactive extraction of *Chlorella sp*. KR-1 in DMC was investigated, and 0.367 g Fatty acid methyl Esters (FAMEs)/g biomass and 0.017 g GlyC/g biomass were obtained at optimized conditions [86]. In Table 3, the research articles about the GlyC production from vegetable oil and DMC are tabulated. Mostly, GlyC production is achieved in solvent-free medium at 60 °C temperature, a reaction time of 48 h, and DMC: Vegetable oil ratio 10:1 or more, under these conditions, more than 90% conversion is reached. Seong et al. investigated enzymatic GlyC production from soybean oil with 6:1 DMC: soybean oil molar ratio at 60 °C, a reaction time of 48 h, and with the solvent tert-butanol, and reached a GlyC yield of 92%. Alternatively, for GlyC production from vegetable oil, the ionic liquid (1-Methyl-3-octyl-imidazolium-hexafluoroborate, Omim[BF_6_]) can also be used [87].

### 5.3. Effect of solvent

Solvents are used in many chemical reactions for mass transfer and to obtain homogeneity of reactor medium; therefore, the solvent type is an important parameter of reaction conditions. The substrates of GlyC, Gly is hydrophilic and DMC is hydrophobic [49]. Gly and DMC are partially miscible to each other [88] so that there is a two-liquid phase building between Gly and DMC [49]. For enzymatic GlyC production, the solubility of Gly in the solvent is important. If Gly is not solubilized, it forms a layer around commercial enzyme, e.g., hydrophilicity support of Novozym 435 [74,89]. This layer building from Gly on enzyme surface reduces the reachability of the enzyme to the substrate (DMC) and Gly conversion to GlyC. Organic solvents during enzymatic GlyC production are applied to improve Gly solubility and to increase reaction medium homogeneity so that efficient mass transfer takes place. According to the literature, for the enzymatic GlyC production from Gly THF [57], acetonitrile [59] and tert-butanol [48,49,58,90] were utilized as solvents (Table 3). Seong et al. used tert-butanol as a solvent for the enzymatic GlyC production from vegetable oil (Table 4) [84]. There are also ionic liquids as an alternative to green solvents listed above (tert-butanol, acetonitrile, etc.) in the production of GlyC from Rapeseed oil. Cushing and Peretti investigated the effect of solvent (e.g., tert-butanol, hexane, and toluene) and the solvent-free condition on Gly conversion. Tert-butanol was the best solvent for the Gly conversion (97%) for 12h reaction time with Novozym 435. The Gly conversion by solvent-free medium was in the same reaction condition was only 49% [90]. Gly can be dissolved both in tert-butanol as in methanol (a by-product of GlyC), so that there is only one phase arisen, the reaction stability is enhanced [80]. Another advantage of tert-butanol is that it is a relatively hydrophilic organic solvent and it increases the enzyme flexibility and can be easily bound to substrates so that the enzyme catalytic activity is improved [39]. Tert-butanol is mostly the preferred solvent for enzymatic GlyC production because it is a nontoxic solvent and results in high Gly conversion (94.85%) using an equimolar amount of DMC and Gly with a reaction time of 14 h [49]. Table 4 shows the solvent effect from different bodies of research. Lanjekar and Rathod reached maximum Gly conversion (94.85%) in 14 h with following reaction conditions: 60 °C reaction temperature, 1.5:1 DMC: Gly molar ratio, 400 rpm, 22% biocatalyst (w/w based on total Gly and DMC amount), tert-butanol as solvent. Because of the inhibitory effect of methanol, the molecular sieve was used [49]. Although the same biocatalyst and same temperature were used in both processes, Gly conversion reached in 14 h was 94.85% in tert-butanol use [49] and 85% in THF use [57]. Based on the analysis of both of those experiments it can be suggested that the solvent addition and DMC:Gly molar ratio directly affects the Gly conversion, and chosen solvent directly affects the reaction time of enzymatic GlyC production.

Although the solvents are used to improve reaction medium homogeneity and enzyme catalytic activity, they are not efficient based on the economic aspect, i.e., their purchasing, recycling, and disposal cost. Lee et al. investigated GlyC production from soybean oil and DMC by Novozyme 435 as well in solvent THF as in solvent-free medium and they also calculated GlyC productivity. The GlyC productivity with solvent was 372.3 $/kg, while the GlyC productivity without solvent was 346.9 $/kg. The GlyC production with solvent needs an extra distillation process for the recovery of solvent [91]. Solvent-free reaction condition is eco-friendly and economic. A solvent-free system with excess DMC is also investigated in the literature [54]. DMC is a nonpolar, nontoxic, and biodegradable (over 90% within 28 days in the atmosphere) solvent [79,80], and it is utilized both as a solvent and as a substrate for enzymatic GlyC production. Lee et al. investigated the enzymatic GlyC production by Novozym 435 in solvent-free media with an excess DMC (10:1 DMC: Gly molar ratio). The reaction runs at 70 °C in 48 h with silica gel addition. A GlyC conversion of 93% was reached [74]. Lee et al. investigated the enzymatic GlyC production from vegetable oil with 15:1 DMC: vegetable oil in solvent-free conditions and reached a Gly conversion of 95.5 % in 48 h at 59.65 °C [56].

### 5.4. Effect of enzyme type and enzyme loading

Enzymes are biocatalysts and lower the activation energy of the reaction by forming an enzyme substrate complex and binding the substrate [68] so that the reaction takes place in a short time. Catalytic transesterification of lipase is encountered often in transesterification reactions, for instance, GlyC production and biodiesel production, in which the Gly as a by-product is formed. Lipases (triacylglycerol ester hydrolase, EC 3.1.1.3) are a kind of enzymatic biocatalyst. Lipases can be extracted from several origins like fungi, bacteria, and yeast and can be used commonly in hydrolysis, esterification/cyclo-esterification, and transesterification reactions because of their excellent chemo-, region-, and stereoscopic selective properties [14,92,93]. Each lipase has different specificity toward its reactants, both Gly/vegetable oil (triglyceride) and DMC. For triglyceride, there are different options as type and/or length of fatty acids, the existence of double bonds and branching. For example, CalB favors fatty acids with the short/medium chain length [14]. *Cal*B and *A.niger* were reported to be effective biocatalysts for the GlyC production, which is listed in Tables 2 and 3. Seong et al. researched the GlyC production with Lipozyme TL IM (*Thermomyces lanuginosus* lipase immobilized on a silica gel) in comparison with Novozyme 435 from Gly and DMC in same reaction conditions (2:1 DMC: Gly molar rate, 60 °C, 200 rpm, %20 enzyme concentration). Although the Gly conversion with Lipozyme TL IM was 20%, the Gly conversion with Novozyme 435 was 4 times higher (80%). Lee and Kim performed a GlyC production from the commercial immobilized enzyme Lipozyme 435 under the reaction conditions 1.63:1 DMC: soybean oil molar rate, 15% biocatalyst loading (% wt based on soybean oil weight), %0.5 water (v/v) and the Gly conversion was 72% [94]. Tudorache et. al. (2012a) has performed an enzyme screening from different organisms (*A. niger*, *Candida antarctica*, *Pseudomonas fluorescens*, *Rhizopus arrhizus*, *Candida cylindracea*, *Pseudomonas cepacia*, *Mucor miehei*, *Aspergillus* sp., *Porcina pancreas*, *Rhizopus niveus*, *Hog pancreas*, *and Thermomyces lanuginosus*), in which Gly conversion and GlyC selectivity (ratio of GlyC moles to sum of the moles of reaction products) under solvent-free condition were compared to each other. It was found out, that the *A. niger* lipase resulted in a Gly conversion of 74% and GlyC selectivity (mol of GlyC to moles of total products) was 80.3%, those were higher than the other biocatalysts (*Candida antarctica* and *Pseudomonas cepacia*). Therefore, *A. niger* was immobilized on a magnetic nanoparticle [60].

The enzyme loading is an important parameter for production cost. Optimum enzyme loading should be chosen so that there should be a balance between enough GlyC concentration and enzyme cost. The reaction rate is increasing with rising enzyme concentration [69] because of higher conversion and rapid enzyme-substrate complex building. At this point, the enzyme comes up to saturation with the substrate and arrives at its maximum velocity [95]. The excess enzyme causes mixing problems by raising viscosity and restricts the mass transfer [96].

Du et al. used CalB lipase, which is immobilized on magnetic organosilica nanoflowers for enzymatic GlyC production. The magnetic organosilica nanoflowers are used because of their hydrophobicity, which encourages the catalytic activity of lipase. The optimum enzyme concentration was 10 g/L because of high Gly yield and GlyC selectivity. With the rising enzyme concentration, the selectivity was reduced because of side product building (GDC and DGTC) [54]. Lee et al. investigated GlyC production from soybean oil and DMC by Novozym 435 with the solvent THF. The best enzyme loading to reach a high GlyC conversion (99.7%) was 116.76 g/L [91]. Tudorache et al. (2012a) investigated the effect of *A.niger* lipase on Gly conversion, GlyC yield, and selectivity. At 12% (w/w) enzyme concentration, Gly conversion and GlyC yield occurred as 81.3% and 60.4%, respectively. With an increase of enzyme loading up to 15% (w/w), Gly conversion and GlyC selectivity remained constant and GlyC yield decreased so that the optimum enzyme loading was decided to be 12% (w/w) [60]. Lanjekar and Rathod investigated enzymatic GlyC production with the commercial immobilized enzyme Novozym 435 in the presence of tert-butanol as solvent. The effect of enzyme loading on Gly conversion was researched. The Gly conversion increased with increasing enzyme loading until 22% (w/w), and above this value, there was a decrease of the Gly conversion observed. For this reason, the enzyme loading of 22% (w/w) was chosen as optimum [49].

### 5.5. Effect of support type and form of the enzyme (free/immobilized)

The immobilization technology is a common technique, which is used to reduce loss of enzymes during biochemical process by reusing of enzymes many times. The immobilized enzyme is preferred more than the free enzyme because of its reusability, easier separation from the product, and greater operational stability [95,97]. The choice of support or matrix is important for the operational stability of immobilization process. The support materials have the ideal properties such as physical resistance to compression, biocompatibility, inertness towards enzymes, hydrophilicity, high surface area, ease of derivization, resistance of microbial attack, and availability at low cost [67]. A literature survey about the biocatalyst type demonstrates that the free enzymes, as well as the immobilized enzymes, are used for the enzymatic GlyC production. *Cal*B and *A. niger* were investigated to be effective biocatalysts for the GlyC production from Gly and vegetable oil, which is listed in Tables 2 and 3. Novozyme 435 (a commercial immobilized enzyme of CalB mostly used immobilized enzyme of CalB, which is immobilized on a macroporous acrylic polymer resin (named Lewatit VP OC 1600) [89]. GlyC production is mostly performed by Novozyme 435 as well from Gly [63,69,90,92–93] (Table 2) as from vegetable oil [56,59,73].

There are also researchers, who used handmade supports to immobilize CalB and *A. niger* lipase for enzymatic GlyC production. Leão et al. investigated an enzymatic GlyC production from handmade immobilized CalB on Accurel MP 1000 support [55]. Gao et al. immobilized CalB on colloidosome support (LP@colloidosome) under the immobilization conditions; 50 ºC, 6 h incubation time, pH 7.5 Tris-HCl buffer (50 mM) and the specific hydrolytic activity of LP@colloidosome was 209.6 U/gsupport and the immobilization yield was 22.08%. The relative activity of the LP@colloidosome, CalB and Novozyme 435 is analyzed after 6 h at different temperatures. At 40 ºC, the retained activity of LP@colloidosome was 80% of initial activity. In contrast to Novozyme 435 and CalB retained approximately 63% of their initial activities. The LP@colloidosome exhibited better thermal stability as free CalB and Novozyme 435 [98]. The reusability of LP@colloidosome was analyzed according to Gly conversion. After 7 times using of this support, 70% of Gly conversion was achieved. By LP@colloidosome the enzymatic GlyC investigated enzyme loading (%6.1 w/w), 60 °C, 1:1 molar ratio of DMC:Gly, and 24 h reaction time, a Gly yield of 64.71% and Gly conversion of 85.20% were obtained. Leão et al. analyzed the enzymatic GlyC production by CalBAcc both with Gly as reactant and compared GlyC production with commercial enzyme Novozyme 435. With the same enzyme loading (20% w/w), 60 °C, 3:1 molar ratio of DMC:Gly, and 48 h reaction time in Brij 76 (surfactant), a GlyC conversion of 70% and a GlyC selectivity of 90% were reached by CalBAcc. The Novozym 435 with the same reaction conditions yielded 51% GlyC conversion and 42% GlyC selectivity [55]. The reason for the low GlyC conversion and selectivity is apparently the external mass transfer limitations consequently low diffusion rate of DMC, which is caused by the accumulation of Gly around Novozyme 435. The Gly can accumulate around the hydrophilicity support of immobilized enzyme as Novozyme 435 [74,89] and cause external mass transfer limitations [99]. Tudorache has immobilized *A. niger* lipase on a magnetic nanoparticle and investigated GlyC production [60–62]. Lee et al. worked with the commercial immobilized form of Cal B (Novozym 435) as well as the free CalB lipase to GlyC production from Gly and DMC, and the GlyC performance was compared to each other. Under the same reaction conditions (with 10:1 DMC:Gly molar ratio, at 60 °C), the free CalB demonstrated a seven-fold higher transesterification rate and Gly conversion than Novozym 435. The reason for this low Gly conversion of Novozym 435 was the coating of Novozym 435 with free Gly because of a two-phase building between Gly and DMC [74]. De Souza et al. immobilized free CalB on different epoxy resin (Purolit^®^ ECR 8205F, Purolit^®^ ECR8214F and Immobead^®^ IB150 P) in 3 mL pH 7 phosphate buffer, 1 g support, 4h, 1mL CalB, and 40 ºC. ECR 8214 showed the best immobilization performance with 57.1% immobilization yield and 2500 U/g specific activity [100]. With ECR8214, the GlyC production was investigated with Gly and DMC. The enzymatic transesterification Gly was performed at 60 ºC, 48 h, 0.18 g Brij76 surfactant, 0.092 g biocatalyst, 3:1 DMC:Gly molar ratio with a Gly conversion of 46% and a GlyC selectivity of 85%. GlyC was also produced from the macaúba oil and DMC by ECR8214 without solvent, 3:1 DMC:macaúba oil molar ratio, 20% (w/w relative oil weight), 60 ºC and 48 h. A Gly conversion of 97% and a GlyC selectivity of 99% were obtained [100]. Lee et al. investigated GlyC production from Gly and DMC with free CalB and Novozyme 435 at 60 ºC with a 10:1 DMC:Gly molar ratio in solvent-free medium. After 48 h, the Gly conversion with Novozyme 435 was 7%, and with CalB, a Gly conversion of approximately 40% was reached. The free CalB showed approximately 6 times more Gly conversion than Novozyme 435. The low mass transfer of substrates to the enzyme Novozyme 435 occurred because of the building of large droplets with Gly around of Novozyme 435 [74].

### 5.6. Reactor type and process classification

Immobilization process yields an easier downstream process and greater process control in different reactor. Enzyme catalysis can be processed as well by batch as continous reactor. Most enzymatic GlyC in Table 2 is produced by batch process. Some researchers made the enzymatic GlyC production in batch and continuous-flow systems. For industrial goals, continuous-flow systems are preferred more than batch reactors because of the availability of process control, higher productivity, and recovery of product quality/yield. There are several types of reactors, which are used for continuous flow. The packed bed reactor (PBR) is one of the most commonly used reactor types for efficient solid-fluid contacting in heterogeneous catalysis as well as biocatalysis. The advantages of the PBR are that (a) biocatalysts can be separated easily [101], (b) it can be operated long term (without losing enzyme activity) and can be scaled up to large industrial processes, [102] (c) it is cost-effective and has higher efficiency than a batch reactor, [103], (d) it does not need high substrate and enzyme ratio compared to batch reactors [104], (e) it presents high surface area and simple-packaged design, [105] (f) enzyme cannot break down due to mechanical shear stress [102,106], and (g) it has shorter reaction time compared to conventional batch reactors [107]. The disadvantage of PBR is that the pH and temperature regulations are not easy [108].

In Table 5, the advantages and disadvantages of batch and continuous reactors are presented. Nascimento et al. designed handmade support from epoxy resins. On this epoxy resin, the combination of lipases from PPL and CalB were immobilized for enzymatic GlyC production from soybean oil and DMC under batch and continuous flow (with a PBR) conditions. In the batch process, a Gly conversion of approximately 95% was reached in 48 h with combined application of (PPL:CalB) on epoxy resin, while in the continuous process, a residence time of 90 min (0.1 mL/min), >99% selectivity, and >99 % conversion were obtained as compared to Novozym 435 (i.e. 88% selectivity). High GlyC productivity (0.16 mg GlyC h^−1^ U^−1^ of biocatalyst) was obtained in the continuous process as compared to the batch process (0.053 mg GlyC h^−1^ U^−1^ of biocatalyst) [109]. Leão et al. investigated an enzymatic GlyC production from CalBAcc. The study was performed using different vegetable oils (soybean oil, palm oil, and macaúba oil) with DMC by CalBAcc and Novozym 435 in continuous-flow (with a PBR) and batch processes. In the batch process, CalBAcc showed better conversion (99% for all vegetable oils) than Novozym 435 (53% soybean oil, 61% palm oil) in 24 h. In the continuous process, CalBAcc reached excellent selectivity (>99%) and high conversion with soybean (99%) and palm oil (93%) of the desired GlyC in a residence time of 176 min.

### 5.7. Effect of reaction time

In an enzymatic transesterification reaction, a short reaction time is desired. With long reaction time, the operating cost increases and the GlyC yield decreases because of side products building with time [39]. Kim et al. investigated the enzymatic GlyC production with Novozym 435 within 28 h in THF solvent and obtained a 99% Gly conversion under reaction conditions: 60 °C reaction temperature, 1:1 DMC:Gly molar ratio, 27% biocatalyst (w/w based on Gly). Here molecular sieve was also used as a scavenger [57]. Du et al. investigated enzymatic GlyC production from magnetic organosilica nanoflowers immobilized CalB lipase. A high selectivity of 94.13% was obtained, and optimum reaction time was 27 h under the following reaction conditions: 20:1 DMC:Gly molar ratio, 10 g/L enzyme amount, 50 °C reaction temperature and in solvent-free medium [54]. Waghmare et al. performed the ultrasonic-assisted stirring enzymatic GlyC production from Gly and DMC with the Novozym 435. In a short reaction time of 4 h, there was a high Gly conversion (99.75%) at 3:1 DMC:Gly molar ratio, 13% Novozym 435 (w/w), 60 °C reaction temperature. With the ultrasound, the reaction time was dropped down to 4 h from 14 h. During the conventional stirring, the Gly conversion was 30% in 4 h, the ultrasound without stirring was given a Gly conversion of 54.30% at the same time. Not only with using ultrasound but also ultrasound combined with stirring, the reaction time was drastically reduced by 10 h [58]. The ultrasonic-assisted process leads to cavitation bubbles collapse between phase borderline from two immiscible solutions so that both layers can be mixed efficiently, the mass transfer limitations reduce, and the reaction rate increases [107,110–112]. Lee et al. performed enzymatic GlyC production with silica gel-coated Gly and DMC by Novozym 435. The advantage of the silica-coated Gly was increase of reaction rate up to 10-fold and reduction of reaction time from 100 h to 48 h [74].

### 5.8. Effect of impurities

W-Gly contains impurities such as methanol, water, soaps, metals (e.g., sodium, potassium, calcium, magnesium, and salts (e.g., phosphates and sulfates) [57,93,95]. These impurities of crude Gly can affect the Gly bioconversion into required products while it inhibits biocatalyst [62,113]. In the literature, there are reports about the GlyC production from W-Gly in solvent-free conditions using different designed immobilized enzymes and free enzymes. This reaction was sensitive to impurities of waste/crude Gly, for instance, methanol and water. Methanol hinders GlyC yield dramatically [114]. The presence of 2% of methanol causes a 20% decrease in GlyC yield [62]. Methanol influences lipase catalytic activity. The short alcohols cause enzymatic denaturation as a result of which the proteins become unstable [62,115]. Jung et al. investigated the effect of water addition from 0.25% to 1.25% (v/v) on the enzymatic GlyC production from Gly and DMC. There was a decrease in Gly conversion from 39.35% to 29.20% observed in 48 h reaction time, 60 °C reaction temperature, 2:1 DMC:Gly molar ratio, 180 rpm stirring, and 75 g/L Novozyme 435 with solvent acetonitrile. With no additional water, the Gly conversion was 77.56% [58]. Min and Lee considered the effect of water addition from 0% to 1% on enzymatic GlyC production from corn oil and DMC (1:10 molar ratio) with the 5% (v/v) Novozym 435. The maximum Gly conversion (99%) was on 0.2% water content (v/v) [116]. If the water content was increased to 1 % (v/v), the Gly conversion falls to approximately 80%. Lipase enzymes require a hydrophobic environment because these enzymes act at lipid-water interfaces [117]. The water content of 11% wt causes a reduction of 30% wt of GlyC production [62]. While a certain amount of water is needed to obtain the lipase activity, high water amount in the reaction medium inhibits the lipase activity [118]. For removing the excess water in the solvent-free system, molecular sieves are used in the literature. However, excess molecular sieves can cause a decrease in reaction rate during the adsorbing of essential water, which is interfered with the enzyme-substrate interaction [54]. For this reason, W-Gly with high contents of methanol and water has to be purified for the lipase-catalyzed production of GlyC. W-Gly from biodiesel production was treated differently. To volatilize residual methanol and water, Gly sample was preheated at 120 °C for approximately 15 min [62]. Tudorache et al. researched Gly conversion and GlyC selectivity in the solvent-free medium of pure and W-Gly (unrefined crude sunflower oil and residual sunflower oil) with DMC. The reaction conditions were 60 °C, 6 h incubation time, %5 (w/w) biocatalyst (on magnetic nanoparticle immobilized *A. niger*), 1:10 Gly/DMC. Pure Gly showed a conversion of 45% and a GlyC selectivity of 92%. The W-Gly from crude sunflowers (29.4% methanol and 28.7% water impurities) occurred with a Gly conversion of 26.8% and a GlyC selectivity of 95%. The residual Gly from sunflower oil sources (6.5% methanol and 29.8% water impurities) showed a Gly conversion of 35.3% and a GlyC selectivity of 90%. The reason for lower Gly conversion of W-Gly from crude sunflower oil is the impurities because of the high amount of methanol. The highest GlyC production was accomplished with pure Gly [82]. In Table 6, studies on water addition under different experimental conditions are presented.

### 5.9. Reusability of enzyme

Reusability plays an important role in the economic aspect as the profitability of enzymatic reaction. The immobilized enzyme offers the possibility of reusability. This property of immobilized enzyme leads to cost-reduction and simplified downstream processing. The immobilized enzyme is preferred more than the free enzyme because of its greater operational stability and easier separation from the product [96]. The reusability of related immobilized enzymes was analyzed with the retention of the initial activity of the enzyme. The initial activity of the enzyme for the first round was taken as 100% [90,116]. The lipase activity can be determined with different methods such as p-nitrophenyl butyrate assay [55] or the esterification activity with oleic acid and 1-butanol to butyl oleate [49,119]. To confirm lipase activity, Gly conversion was also measured after every cycle [49,74]. After the reaction, the immobilized enzyme should be washed to remove substrate/product or solvent residue. In the literature, it was reported that the commercial Novozym 435 was washed with acetone several times and the washed enzyme was dried out in a desiccator [116,119]. Novozym 435 maintained 80% of this initial activity after seven times recycling so that this enzyme can be used more than seven times [116]. In Table 7, the reusability of enzyme, its washed chemicals, and the residual activity are presented. A literature review in the reusability of immobilized enzyme, which is used in GlyC production reveals that the reusability of Novozyme 435 is given mostly 4–7 times [54, 64, 76, 87, 90, 111]. Cushing et al. investigated the reusability of Novozyme 435 with using of tert-butanol as washed chemicals. After 5 times reusability of Novozyme 435, the residual activity was 85% [90]. Min and Lee researched the reusability of Novozyme 435 until 7 times with a residual activity of 80%, which was washed with acetone as solvent [116]. Tudorache et al. found a reusability up to 20 times during GlyC production without loss of catalytic activity [120].

## 6. Conclusions and perspectives

The transesterification with DMC provides high chemical reactivity in comparison to another carbonate derivate as the reactant for GlyC synthesis. GlyC can be produced by a chemical- or biochemical catalyst. The enzymatic synthesis offers mild reaction conditions (temperature, pressure, and solvent-free) and high GlyC selectivity; therefore, it is a great alternative to chemical synthesis for the GlyC synthesis from Gly/vegetable oil and DMC. In this review, the enzymatic synthesis route of GlyC from Gly/vegetable oil with DMC is presented. GlyC can be produced by pure/W-Gly and vegetable oil with DMC. The parameters that affect GlyC production were also described in detail. The increasing global warming warrants ecofriendly oxygenated fuel additives such asGlyC, which is a potential fuel additive because of its high oxygen content. Another alternative, the GlyC from vegetable oil with DMC is produced during biodiesel production. With this synthesis route, the risk with methanol and Gly inhibition of the commercial enzyme (for example Novozym 435) is reduced and this Gly-free biodiesel with GlyC can be directly used as fuel, and this process can be easily integrated to a biodiesel plant. Thus, the integration of GlyC production from vegetable oil will directly reduce the cost of purification step of Gly, and Gly production as by-product will be prevented. Furthermore, in the future, the drastic demand in electronics and automotive industries (electrical vehicle) will increase the use of environmentally friendly GlyC, which is used as additive in production of lithium-ion batteries.

## Figures and Tables

**Table 1:** Advantages and disadvantages of different carbonate derivates used in transesterification of GlyC.

Carbonate derivate	Advantage	Disadvantage
DEC (By-product: ethanol)	Nontoxic for lipase With increasing chain lengths of substrates, more recognition and better conformation to lipase active site [90] Versatile chemical and ecofriendly High chemical reactivity High energy consumption during separation process is not required [50]	Lower GlyC conversion than DMC [82]
EC (By-product: ethylene glycol)	Low reaction temperature is required [33]	EC is expensive [50] Because of the high boiling point (261 °C) of ethylene glycol, high purification cost is required [49]
DMC (By-product: methanol)	No enzyme deactivation with DMC [116] High chemical reactivity Lower boiling point (90 °C) Nontoxic and biodegradable High energy consumption during separation process is not required [50]	Azeotrope forming with methanol [40]

**Table 2:** Lipase catalysis conditions for enzymatic GlyC synthesis with DMC and Gly.

Catalyst	Catalyst loading (wt, %)^a^,^b^	Reactants mole ratio DMC:Gly	Source	Solvent	Temperature [°C]	Reaction time [h]	GlyC Yield (Y) Conversion (C) Selectivity (S) [%]	Reference

Novozym 435	27 a	1:1	Gly	THF	60	28	C = 99	[57]

Cal B (free) Novozym 435	5 a	10:1	Gly	-	70	48	C = 93	[74]

*A. niger* (free)	12 a	10:1	Gly	-	60	4	C = 74 S = 80.4	[60]

*A. niger* (immobilized on magnetic nanoparticle)	5 a	10:1	Gly/W-Gly from residual and different oil	-	60	6	Y = 55	[62]

*A. niger* (immobilized on magnetic nanoparticle)	5 a	10:1	Gly/W-Gly from crude and residual sunflower oil	-	60	6	C_Gly_ = 45 S_Gly_ = 92 C_w-Gly,cru_ = 26.8 S_w-Gly,cru_ = 95 C_w-Gly,res_ = 35.3 S_w-Gly,res_ = 90	[82]

*A. niger* (immobilized on magnetic nanoparticle)	5 a	10:1	W-Gly from different raw material	-	60	6	Y = 95	[118]

Novozym 435	13^b^	3:1	Gly	Tert-Butanol	60	4	C = 99.75	[58]

*A. niger* (immobilized on magnetic nanoparticle)	5^a^	10:1	Gly	-	60	6	C = 87.7	[61]

Novozym 435	22^b^	1.5:1	Gly	Tert-Butanol	60	14	C = 94.85	[49]
Novozym 435	75 c	2:1	Gly	Aceto-nitrile	60	48	C = 96.25	[59]

Cal B LypozmeTL 100L EversaTransforma (free enzymes) Novozym 435	3	3:1	Gly	Tert-Butanol	60	100	Y = 81	[48]

Novozym 435 CalBAcc (*C. antarctica* immobilized on AccurelMP1000)	20	2:1	Gly	-	60	48	C = 80	[55]

Cal B Lipase (immobilized on magnetic organosilica)	10^c^	20:1	Gly	-	50	27	S = 94.13	[54]

Novozym 435	5^a^	10:1	Gly	Tert-Butanol	50	12	C = 97	[90]
LP@ Colloidosome	6.1 b	10:1	Gly	Tert-Butanol	50	24	C = 85.2 Y = 64.7	[98]
ECR8214	15 a	0.3:1	Gly	-	60	48	C = 46 S = 85	[100]
*A. niger* (immobilized on magnetic nanoparticle) (Poly-Gla)	-	10:1	Crude Gly	-	60	6	C = 70 S = 80	[114]
*A. niger* (immobilized on magnetic nanoparticle (Gla)	5^a^	10:1	Crude Gly	-	60	6	C = 61 S = 90	[120]

a (wt, %, based on glycerol weight)

b (wt, %, based on glycerol and DMC weight)

c (g/L) (Gla: gluteraldehyde (cross-linker))

**Table 3:** Lipase catalyzed conditions for enzymatic GlyC synthesis with DMC and vegetable oil.

Catalyst	Catalyst loading (g/L)^c^ (wt,%)^d^	Reactants mole ratio DMC: Vegetable oil	Source	Solvent	Temperature [° C]	Reaction time [h]	GlyC Yield (Y) Conversion (C) Selectivity (S) [%]	Reference

Novozym 435	25 c	15:1 )	Soybean oil (SO)	-	59.65	48	C = 95.5	[56]

Cal B / PPL (immobilized on epoxy Resin) Novozym 435	20 d	10:1	SO	-	60	48	C = >99 S = 99	[109]

Novozym 435 CalBAcc	20 d	10:1	SO Palm oil (PO) Macaúba oil (MO)	-	60	48b* ~3c*	C_(SO), (PO)_,_(MO)_: 99 b* S_(SO),(PO)_,_(MO)_:>99 b* C_(SO)_:> 99 b* C_(PO)_: 93 c* C_(MO)_: 73 c* S_(SO),(PO)_,_(MO)_:>99 c*	[55]

Novozym 435	10 d	10:1	Corn oil	-	60	15	Y = 62	[116]

Novozym 435 Lipozyme RM IM Lipozyme TL IM *A. niger* *P. fluorescens* *P. camemberti* *R.oryzae* (free enzymes)	100 c	6:1	SO	Tert-butanol	60	48	Y = 92	[84]

Novozym 435	20 ^n/a^	10:1	SO	-	60	48	C = 92.1	[73]

Novozym 435	20 ^n/a^	3:1	Rapeseed oil	[Omim] [BF_6_]	n/a	48	C = 73.87	[87]
Novozym 435	116.76^c^	9.27:1	SO	THF	52.56	n/a	C = 99.7	[91]
Lipozyme 435	15 d	1.63:1	SO	-	n/a	60	C = 72	[94]
ECR8214	20 d	10:1	MO	Brij76	48	60	C = 97 S = 99	[100]
Calb (immobilized on celite)	10 c	-	Microalgal oil	-	4	50	Y = 2.7	[76]

c (g/L)

d (wt, %, based on vegetable oil weight)

n/a: No information available

b* Batch reaction

c* Continuous flow reaction condition

**Table 4:** Effect of solvent using for GlyC production.

Gly conversion [%]		Reaction conditions	References
With solvent	Solvent-free		
Tert-butanol 97	49	50 °C, 12 h, 5% (wt ) Novozym 435, 2:1 DMC:Gly	[90]
Tert-butanol ~30	≃ 3	60 °C, 6 h, n/a (wt) Novozym 435, 1:1 DMC:Gly, 3.33 g molecular sieves	[49]

**Table 5:** Advantages and disadvantages of batch and continuous reactors.

Reactor type	Advantage	Disadvantage
Batch	It is operable for the cheap free enzyme [106]	It needs high substrate and enzyme ratio Loss of enzyme activity Immobilized enzyme may be destroyed in the recovery process [106] High operation costs [103]
Continuous (CSTR)	Better process control Available to scaled-up to large industrial process [102] Higher productivity Recovery of product quality/yield Shorter reaction time [107] Easily separated biocatalyst [101]	Not easy pH and temperature regulation [108]

**Table 6:** Experimental conditions of research with water addition (reactants: pure glycerol and DMC).

Water content range [v/v %]	Optimum water content [v/v %]	References

0–1.5	0.7	[59]
-	0.82	[55]
0.75–1	0.2	[56]
-	0.7	[109]
0–3	0.7	[73]

v/v : Volume of water/total reactant volume

**Table 7:** Reusability of the enzyme, its washed chemicals, and residual activity.

Reusability	Washed chemical	Residual activity [%]	Enzyme	References

4	-	-	Novozyme 435	[74]
6	Acetone	-	Novozyme 435	[58]
10	Water and phosphate buffer	-	*A.niger* (I )	[61]
6	Acetone	-	Novozyme 435	[49]
7	DMC	79	CalB (I)	[54]
5	Tert-butanol	85	Novozyme 435	[90]
7	Acetone	80	Novozyme 435	[116]
5	-	-	Novozyme 435	[87]
20	-	-	*A.niger* (I)	[81][120]

I: Immobilized
